# Reliability and validity of brain‐gastric phase synchronization

**DOI:** 10.1002/hbm.26436

**Published:** 2023-08-01

**Authors:** Gidon Levakov, Shira Ganor, Galia Avidan

**Affiliations:** ^1^ Department of Cognitive and Brain Sciences Ben‐Gurion University of the Negev Beer‐Sheva Israel; ^2^ Department of Psychology Ben‐Gurion University of the Negev Beer‐Sheva Israel

**Keywords:** electrogastrography, fMRI, interoception, resting‐state, stomach

## Abstract

Recent studies have reported that various brain regions, mainly sensory, unimodal regions, display phase synchronization with the stomach's slow (0.05 Hz) myoelectrical rhythm. These gastric–brain interactions have broad implications, from feeding behavior to functional gastrointestinal disorders. However, in contrast to other interoceptive signals (e.g., heart rate) and their relation to the brain, little is known about the reliability of these gastric–brain interactions, their robustness to artifacts such as motion, and whether they can be generalized to new samples. Here we examined these aspects in 43 subjects that had undergone multiple runs of concurrent electrogastrography (EGG), brain fMRI, and pulse oximetry. We also repeated all analyses in an open dataset of a highly sampled individual. We found a set of brain regions that were coupled with the EGG signal after controlling for non‐grey matter (GM) signals, head motion, and cardiac artifacts. These regions exhibited significant overlap with previous work. However, we also showed that prior to confound regression, the spatial extent of the gastric network was largely overestimated. Finally, we found substantial test–retest reliability in both the brain and the gastric signals when estimated alone, but not for measures of gastric‐brain synchrony. Together, these results provide methodological scaffolding for future research into brain–stomach interactions and for a better understanding of the role of the gastric network.

## Graphical abstract

In a study of 43 subjects who underwent concurrent electrogastrography (EGG), brain fMRI, and pulse oximetry, it was found that a set of brain regions were coupled with the EGG signal after controlling for non‐grey matter (GM) signals, head motion, and cardiac artifacts. The spatial extent of the gastric network was found to be consistent with prior work. Test‐retest reliability in both the brain and gastric signals was found to be substantial when estimated alone, but not for measures of gastric‐brain synchrony.

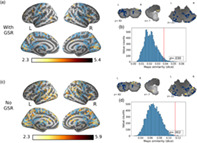

## 1 INTRODUCTION

The brain and the gastric system interact through multiple physiological and anatomical routes (Mayer, 2011). Recently, several studies have reported the existence of a wide network of brain regions that are specifically coupled with the stomach's slow rhythmic electrical signal (Choe et al., 2021; Rebollo et al., 2018; Rebollo & Tallon‐Baudry, 2022). These regions were mainly composed of sensory and motor cortical areas that showed lagged synchronization with the gastric electrical activity. Similar to the cardiac rhythm, this activity is generated by internal “pacemakers,” the intestine cells of Cajal, that coordinate the peristaltic waves that move through the stomach (Grundy & Brookes, 2011). These gastric–brain interactions have important implications for functional gastrointestinal disorders, eating disorders, emotion, and cognition (Drossman, 2016; Mayer, 2011). However, research into these interactions still lags behind current knowledge on other interoceptive signals, such as those arising from the heart (Coll et al., 2021).

Since the first report of the gastric network (Rebollo et al., 2018), additional research has advanced our understanding of the anatomical and functional characteristics of brain‐gastric coupling. For example, the location of the gastric network within unimodal compared to multimodal cortical regions (Rebollo & Tallon‐Baudry, 2022) and its modulation by stimulation of the vagus nerve (Müller et al., 2022). These previous studies used a similar approach, measuring phase synchronization between the stomach activity as recorded using cutaneous electrogastrography (EGG) and brain functional magnetic resonance imaging (fMRI) recordings.

Both fMRI (Caballero‐Gaudes & Reynolds, 2016) and EGG (Parkman et al., 2003) recordings are highly susceptible to noise that arise, for example, from motion or cardiac activity. Prior research has provided partial solutions for these artifacts by correcting for cardiac and respiratory‐induced noise using RETROICOR (Müller et al., 2022; Rebollo & Tallon‐Baudry, 2022), a method for retrospective correction of physiological motion effects (Glover et al., 2000). However, other corrective measures typically employed in functional connectivity studies, such as the inclusion of motion parameters as a nuisance regressor (Ciric et al., 2017), have not been applied (see Table S1). Thus, the effect of these confounds on gastric‐brain synchrony is still not clear. Moreover, the extent to which gastric‐brain synchrony is consistent within subjects, that is, its test–retest reliability, has not been tested before. These outstanding questions should be considered before addressing broader questions pertaining, for example, to the role of the gastric network in physiology and behavior.

The goal of the current work was to examine the validity and reliability of phase synchronization between the stomach and the brain. Specifically, we focused on the following questions: (1) Whether previous findings of the gastric network would generalize to a new sample following strict control of non‐neuronal confound. (2) What is the specific effect of various confounds on estimating gastric‐brain synchrony? and (3) Do current measures of the gastric network exhibit test–retest reliability? To answer these questions, we acquired a sample of 43 subjects with repeated concurrent measures of EGG and fMRI. Additionally, we repeated all analyses in an open dataset containing a single, highly sampled individual. We first demonstrated that previous results of the gastric network could be generalized to an analysis pipeline that includes a rigorous correction for motion as well as additional artifacts. We examined whether various confounds are synchronized with the EGG signal and found such synchrony for non‐GM fMRI signal and head motion. The global signal was also found to correlate with the EGG signal. Moreover, this synchrony was correlated with the extent of brain‐wide gastric network estimation obtained when motion artifacts were not removed. Finally, we measured the test–retest reliability of the EGG‐brain synchrony and found that it was remarkably low compared to measuring the reliability of the fMRI and the EGG signals separately. We advocate that a careful examination of the generalizability, validity, and reliability of brain–visceral interactions, as examined here, will pave the way for future studies which will enable a better understanding of the nature of gastric–brain interactions.

## 2 MATERIALS AND METHODS

### 2.1 Participants

Fifty‐nine subjects participated in the current study. Subjects were recruited after attending one of two Electrogastrography experiments in the Visual & Interoceptive perception lab at Ben Gurion University of the Negev. Inclusion criteria included having an EGG signal with sufficient quality in the lab experiment (see Section 2.5), BMI between 18 and 25, absence of gastric‐related disorders, and compliance with all MRI safety regulations. All subjects provided informed consent, and the study was approved by the Human Subject Committee of the Soroka Medical Center. Exclusion criteria included poor EGG quality or excessive head motion within the scanner (see Methods 2.5 and 2.3). A total of 84 runs of 43 subjects were included in the following analyses (27 female, 16 male; mean age 25.19 ± 3.35). In addition, we validated all the results using the Concurrent Electrogastrography/Resting Brain fMRI (CERB) highly‐sampled‐individual data resource (https://www.nitrc.org/projects/cerb_2020). The data contained 19 sessions of two 15‐minute runs of concurrent EGG and fMRI of a single individual (Male, age 58). The participant provided informed consent, and the study was approved by the Johns Hopkins Medicine Institutional Review Board. Additional information on acquiring the EGG and MRI data is provided in the relevant publication (Choe et al., 2021).

### 2.2 MRI acquisition

MRI scans were conducted at the Soroka University Medical Center (SUMC), Beer Sheva, Israel. Participants were scanned in a 3T Philips Ingenia scanner (Amsterdam, The Netherlands) equipped with a standard head coil. Before the MRI sessions, all participants were instructed to refrain from eating for 3 h and drinking for half an hour prior to scanning. Each session included a 3D T1‐weighted anatomical scan and two concurrent electrogastrography and resting state‐fMRI runs of 15 min each (*n* = 54) or three 10 min runs (*n* = 5). Before each resting‐state run, subjects were instructed to remain awake with their eyes open, lie still, and watch a blank screen. fMRI BOLD contrast was acquired using the gradient‐echo Echo‐Planar imaging sequence with a parallel acquisition (SENSE: factor 2.4). Specific scanning parameters were as follows: whole‐brain coverage 44 slices (3 × 3 × 3 mm^3^), transverse orientation, 3 mm thickness, no gap, TR = 2000 ms, TE = 25 ms, flip angle = 77°, FOV = 192 × 192 (RL × AP) and matrix size 64 × 64 (RL × AP). High‐resolution anatomical volumes were acquired with a T1‐weighted 3D pulse sequence (1 × 1 × 1 mm^3^, 150 slices).

### 2.3 MRI preprocessing

Preprocessing of structural and functional imaging was conducted using fMRIprep 20.0.6 (Esteban et al., 2019), which is based on Nipype 1.4.2 (Gorgolewski et al., 2011). Parts of this section are adopted from the fMRIprep preprocessing report. The T1‐weighted (T1w) image was corrected for intensity nonuniformity with N4BiasFieldCorrection (Tustison et al., 2010), distributed with ANTs 2.2.0 (Avants et al., 2008), and used as T1w‐reference. The T1w‐reference was then skull‐stripped with a Nipype implementation of the antsBrainExtraction.sh workflow (from ANTs), using OASIS30ANTs as the target template. Brain tissue segmentation of cerebrospinal fluid (CSF), white matter (WM), and grey matter (GM) was performed on the brain‐extracted T1w using fast (FSL 5.0.9; Zhang et al., 2001). Brain surfaces were reconstructed using recon‐all (FreeSurfer 6.0.1; Dale et al., 1999), and the brain mask estimated previously was refined with a custom variation of the method to reconcile ANTs‐derived and FreeSurfer‐derived segmentations of the cortical grey‐matter of Mindboggle (Klein et al., 2017). Volume‐based spatial normalization to the MNI152NLin6Asym space (Evans et al., 2012) was performed through nonlinear registration with antsRegistration (ANTs 2.2.0), using brain‐extracted versions of both T1w reference and the T1w template.

Each of the resting‐state fMRI runs was preprocessed as follows: First, a reference volume and its skull‐stripped version were generated using a custom methodology of fMRIPrep. Susceptibility distortion correction (SDC) was omitted. The BOLD reference was then co‐registered to the T1w reference using bbregister (FreeSurfer), which implements boundary‐based registration (Greve & Fischl, 2009). Co‐registration was configured with six degrees of freedom. Head‐motion parameters with respect to the BOLD reference (transformation matrices and six corresponding rotation and translation parameters) were estimated prior to any spatiotemporal filtering using mcflirt (FSL 5.0.9; Jenkinson et al., 2002). BOLD runs were slice‐time corrected using 3dTshift from AFNI 20160207 (Cox & Hyde, 1997). The BOLD time series, including slice‐timing correction, were resampled onto their original, native space by applying the transforms to correct for head‐motion. The BOLD time series were resampled into the MNI152NLin6Asym standard space. The following time series were regressed out: the global signal (GS), framewise displacement (FD; Power et al., 2014), six motion estimates, their derivatives and their squares, and six component‐based noise corrections from the CSF and WM (aCompCor Behzadi et al., 2007). In addition to the main preprocessing pipeline, we had three additional versions: (1) A version that included all the regressors of the main version except the global signal. (2) A version that included all the regressors of the main version, including the global signal, in addition to six retrospective corrections of physiological cardiac effects (RETROICOR) components. (3) A version that included only the CSF as a regressor (“minimally preprocessed”). The third version was created to examine the effect of confound regression on the estimated brain‐gastric synchrony (Section 3.2). All versions were spatially smoothed (FWHM = 3 mm), and each voxel was bandpass filtered around the gastric peak using the same parameters of the EGG filtering (see Section 2.5). Subjects with mean FD that was higher than two standard deviations from the group mean were excluded. This resulted in the exclusion of 5 runs out of the total 122 runs.

### 2.4 Cardiac and gastric signal acquisition

EGG recordings were conducted both in the lab and within the MRI scanner using an MRI‐compatible BIOPAC amplifier (EGG‐100C, BIOPAC Systems Inc.). In both settings, subjects were placed in a supine position and were instructed to avoid any movement. Following skin preparation that included cleaning with alcohol pads and administering a small amount of conductive gel at each electrode location, standard cutaneous electrodes (EL503, EL508 BIOPAC Systems Inc.) were attached to the subject's abdomen following previous studies (Figure S1; Rebollo et al., 2018; Wolpert et al., 2020). Four pairs of electrodes were placed on the abdomen, and a ground electrode was located on the hip bone to record four channels. The EGG amplifier was used with a sample rate of 5000 Hz, a gain of 5000, a high pass filter of 0.005 Hz, and a low pass filter of 1 Hz. The cardiac signal was recorded using an MRI‐compatible pulse oximeter (SpO_2_, Philips Inc.) to avoid gradient artifacts. The pulse probe was placed on the left index finger, and the pulse was recorded with a sample rate of 500 Hz.

### 2.5 Cardiac and gastric signal preprocessing

EGG preprocessing was conducted using an in‐house code (https://github.com/GidLev/brain_gastric_synchronization_2023) and was based on previous work (Rebollo et al., 2018). First, the recorded signal was downsampled from 5000 to 10 Hz. Next, we selected the recording channel that exhibits the maximal gastric activity, that is, the channel with the largest spectral power peak at the typical gastric frequency range of 0.033–0.066 Hz. Spectral power estimation was done using Welch's method with a 200 s window and a 150 s overlap. The selected channel was filtered around the peak gastric frequency (±0.015 Hz) using a finite impulse response bandpass filter (MNE‐python: firwin2). Recording quality was assessed in line with a previous study (Wolpert et al., 2020) using all channels before bandpass filtering. In brief, EGG signal quality was assessed using three criteria: a clear peak within the normal EGG range (0.033–0.066 Hz), consistency in the gastric peak across channels, and consistency in the gastric peak across runs. According to these criteria, the EGG data was assigned to one of three quality levels (1—high, 2—medium, and 3—low). In line with these criteria, we selected the channel with the second‐largest spectral power peak in cases it displayed better quality. All samples assigned a level 3 quality were excluded. As a result, 29 of 122 runs were discarded, an exclusion rate that is comparable with previous work (21 of 117 discarded; Wolpert et al., 2020). Subject reliability was assessed only for subjects with two runs that were not excluded due to poor EGG or BOLD quality resulting in a higher exclusion rate (36 of 59 subjects excluded). Figure S2 depicts the spectrogram of all subjects, selected channels, and assigned quality levels. Pulse oximetry was taken to assess the possible effect of the cardiac signal on gastric‐brain synchrony. The cardiac peaks were detected (Systole: ppg_peaks; Legrand & Allen, 2022) and used to create six components using the retrospective correction of physiological motion effects (RETROICOR) model (Glover et al., 2000). Pulse oximetry was recorded for 93 of 122 runs.

### 2.6 Assessing brain‐gastric synchrony

The analysis of brain‐gastric synchrony at the level of an individual run and at the group level (Section 2.7) was adopted from Rebollo et al. (2018). Brain‐gastric synchrony was assessed using the phase‐locking value (PLV) (Lachaux et al., 1999) separately for each brain voxel. The PLV quantifies whether a consistent phase lag exists between two signals, and its value ranges between zero (no synchrony) and one (perfect lagged synchrony). First, the first 15 volumes (30 s) of the brain and gastric time series were excluded. Next, the phase of both signals was computed by applying the Hilbert transformation and taking the angle of the resulting signal. Then, the gastric signal was down‐sampled from 10 Hz to the BOLD frequency 0.5 Hz. Finally, the absolute value of the mean difference in the angle of both signals across time was computed as in Equation 1:

$${\mathrm{PLV}}_{x,y}=\left|\frac{1}{T}\sum_{t=1}^{T}\left(e^{i\left(\theta \left(x_{t}\right)-\theta \left(y_{t}\right)\right)}\right)\right|$$

Here, *x* and *y* are the gastric and brain voxel time series, and *T* is the number of time points. The PLV could be affected by multiple factors, for example, by the extent that it contains different frequencies. Hence, PLV is typically compared to a null model obtained here by shifting the gastric signal in time. We created circular shifts (numpy.roll; Harris et al., 2020) of the gastric signal by at least ±60 s, resulting in 360 null signals for 15 min recordings and 210 for 10 min recordings. Next, the PLV of each voxel with the null signals was computed, and the median of all null PLVs was taken (“median null PLV”). Finally, the difference between the empirical PLV and the median null PLV (“PLV‐delta”) was averaged across the two runs and taken as the final subject‐level measure of gastric‐brain synchrony.

### 2.7 Group‐level assessment of brain‐gastric synchrony

To identify brain regions that are synchronized with the gastric signal at the group level, we tested the difference between the empirical and the time‐shifted PLV values across subjects. First, the PLV and the median null PLV maps were each averaged across runs of the same individual to create subject‐level maps. Next, a paired *t*‐test between the brain maps of the PLV and the median null PLV was conducted. To identify significant voxels while controlling for multiple comparisons, we used a nonparametric permutation test (FSL: randomize; Winkler et al., 2014). In brief, the threshold *t*‐value maps (*t* > 2.3) were compared to a distribution obtained by shuffling the empirical and null maps within subjects 10,000 times. The significance of the clusters was determined by comparing their mass to the null distribution (*p* < .05, family‐wise error corrected). A cluster's mass is the sum of its *t*‐values, a statistic that incorporates both cluster size and intensity values.

### 2.8 Measuring gastric‐brain synchrony and generalization of previous work following motion correction

The main analysis, depicting the brain regions that demonstrate a significant PLV with the EGG signal, was conducted on the data resulting from the main preprocessing pipeline (Section 2.3), but all analyses were replicated with a version that also included a correction for the cardiac signal (Supporting Information 8.2). All surface and volume plots were created with Pysurfer (https://github.com/nipy/PySurfer) and Nilearn (Abraham et al., 2014). The spatial similarity to Rebollo and Tallon‐Baudry (2022) gastric network was assessed using Dice similarity. A null model to determine the significance level of the similarity was derived by creating 10,000 spatially permuted versions of the Rebollo and Tallon‐Baudry (2022) gastric network (Alexander‐Bloch et al., 2018). Briefly, significant voxels of both maps were projected to a sphere (Neuromaps: mni152_to_fsaverage; Markello et al., 2022). Then, for each permutation, a random rotation was applied to the sphere of the right hemisphere and then the opposite rotation to the left hemisphere with respect to the sagittal plane to preserve hemispheric symmetry (Neuromaps: alexander_bloch). Finally, the dice similarities of the gastric network obtained in the current study with the rotated null maps were computed to create a null distribution.

### 2.9 The relation of motion and additional confounds with brain‐gastric synchrony

The PLV of the EGG signal with each of the confounds was compared using a paired *t*‐test to a median null PLV created by shifting the EGG signal in time, as described in Section 2.7. The correlation of whole‐brain PLV‐delta with EGG‐confounds synchrony was assessed with Spearman's correlation. Whole‐brain PLV was taken as the mean delta PLV with the brain mask (FSL; Zhang et al., 2001). EGG‐confounds synchrony was quantified using the PLV. These correlations were computed twice, once with the minimal preprocessing BOLD data and once with the main, strict, confound regression BOLD data (Section 2.3). The observed brain‐gastric synchrony for the two versions was quantified by the difference in the t‐values (Section 2.7) within the brain mask and the number of significant voxels.

### 2.10 Within‐subject reliability of the EGG and brain signals and the synchrony among them

To examine the within‐subject reliability of brain‐gastric synchrony, we correlated it across the first and second runs of each subject. This analysis was conducted only for subjects with two runs that were not excluded due to poor EGG or BOLD quality (*n* = 23; 46 runs). Brain‐gastric synchrony was assessed using the average PLV‐delta within the brain and GM mask and the mean PLV within each of Schaefer's 100 atlas nodes. EGG peak frequency reliability was evaluated using Pearson's correlation between the first gastric recording in the lab and the first gastric recording in the MRI. Functional connectivity reliability was measured as the correlation of all unique edges between two runs of the same subject compared to runs of two different subjects. The difference of the between compared to the within correlations was assessed using an independent *t*‐test.

## 3 RESULTS

### 3.1 Measuring gastric‐brain synchrony and generalization of previous work

**FIGURE 1:**
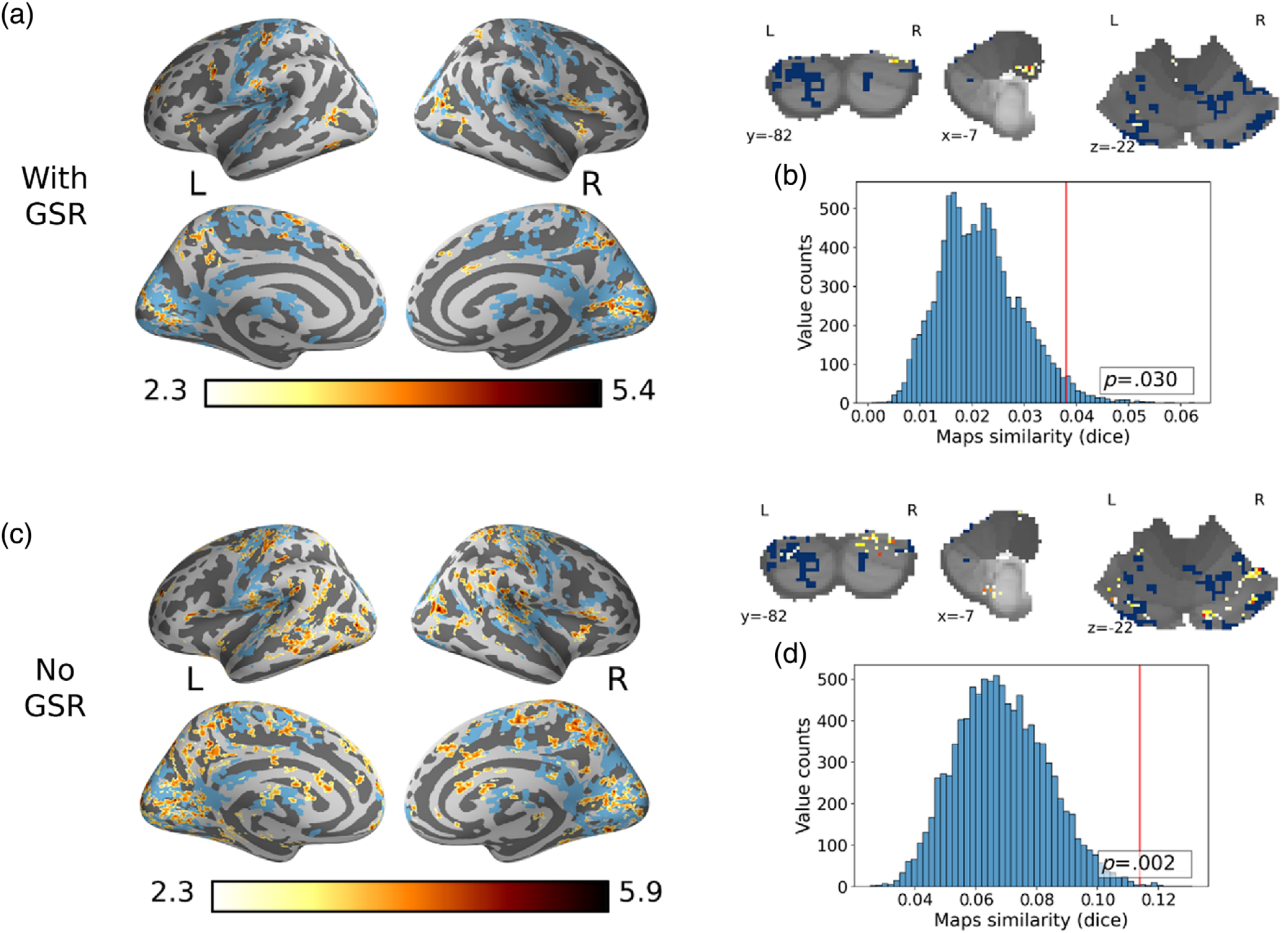
Generalizability of the gastric‐brain network. The results are shown following strict confound control with (top panel) and without (bottom panel) global signal regression (GSR) from the brain signal. Clusters composing the gastric network are depicted on an inflated brain surface and on coronal, sagittal, and axial cerebellum slices (a,c). Significant clusters obtained from the current study are presented in yellow to red colors representing voxels' *t* values. Significant clusters from Rebollo and Tallon‐Baudry (2022) are presented in blue. (b,d) A histogram of the null distribution of the Dice similarity values obtained from the map of the present study and the spatially permuted map of Rebollo and Tallon‐Baudry (2022). The vertical red line represents the empirical similarity.

We first set out to examine the generalizability of previous findings of the gastric network. To do so, we calculated the phase‐locking value (PLV) between the EGG signal and each voxel for all subjects and runs following strict confound control with and without global signal regression (GSR) (Section 2.3). The empirical PLV was compared to the median of a PLV null distribution generated by shifting the EGG signal in time. We identified clusters that were significantly synchronized with the EGG signal by comparing the empirical and null PLV while controlling for family‐wise error based on the clusters' mass. The significant clusters are presented on an inflated brain surface and the cerebellum volume (Figure 1). These clusters include the occipital lobes and precuneus, the bilateral insula, and the pre and postcentral gyri. Next, we compared the spatial similarity between our results and those reported by Rebollo and Tallon‐Baudry (2022). This spatial similarity was assessed using Dice similarity and was compared to the similarity of our map with 10,000 spatially permuted versions of the Rebollo and Tallon‐Baudry (2022) map. The empirical similarity was significantly larger than the null distribution (GSR: Dice = .035, *p* = .03; No GSR: Dice = .114, *p* = .002). These results were replicated after the removal of the cardiac signal in a sub‐sample of subjects with pulse oximetry data (*p* = .02; Supporting Information 8.1). Utilizing the PLV of each subject's brain with the stomachs of other participants as a null model (Choe et al., 2021), also yielded a significant cluster within the gastric network, albeit of reduced volume (Supporting Information  8.2). Finally, we conducted the same analyses in a dataset of a highly‐sampled individual. We found significant clusters in the right precuneus and paracentral lobule. We then compared the obtained map to Rebollo and Tallon‐Baudry (2022) and found no evidence of similarity (*p* = .42; Supporting Information 8.3). We note that the lack of similarity might result from using a single subject compared to a large group.

### 3.2 The relation of motion and additional confounds to brain‐gastric synchrony

**FIGURE 2:**
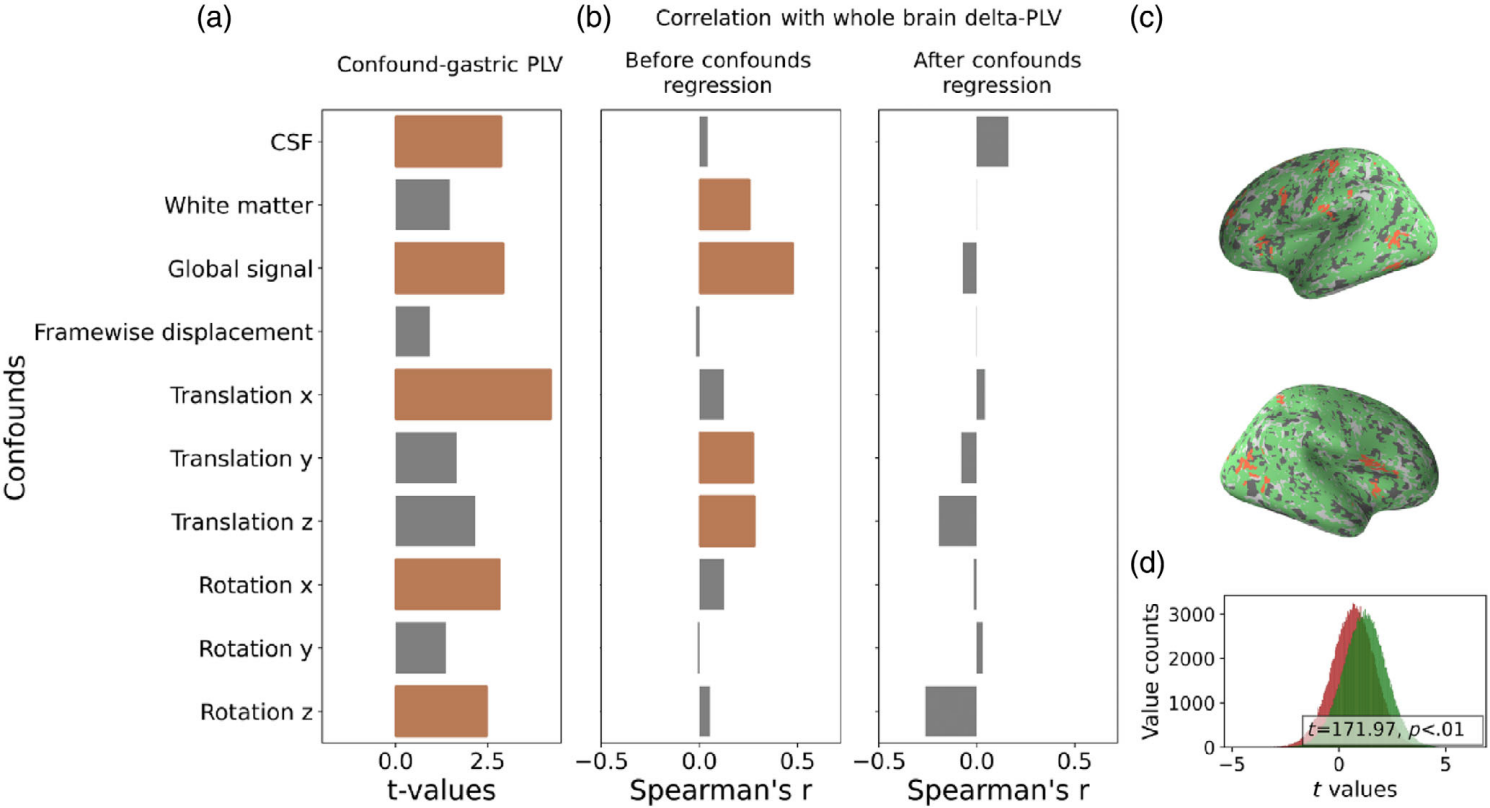
The effect of potential confounds on the gastric‐brain estimated synchrony. (a) Synchrony between confounds and the EGG signal. Bars represent the paired *t*‐test values between the PLV obtained with the empirical compared to the permuted EGG signal. (b) The correlation of EGG‐confounds synchrony with the extent of brain‐wide gastric‐brain synchrony. The correlations are presented before (left) and after (right) confounds regression from the BOLD signal. In all bar plots, brown‐colored bars were significant following a correction for multiple comparisons (*p* < .05, FDR corrected). (c) Significant voxels before (green) and after (red) confound regression. Note the brain‐wide spread of significant voxels prior to cofound control (confounded: %19.89 of the GM, cleaned: 1.16% of the GM). (d) The distribution of whole brain *t*‐values before (green) and after (red) confound regression.

fMRI is highly susceptible to noise of non‐neuronal origins, such as head motion or cardiac‐induced pulsation (Caballero‐Gaudes & Reynolds, 2016). Although some of these confounds might also affect the EGG signal, motion parameters were not added as nuisance regressors in previous work (see Table S1). Here we assessed whether these confounds were synchronized with the EGG signal and examined their possible contribution to brain‐wide synchrony with the EGG signal. We assessed nine non‐GM signal in addition to the global signal as possible confound, although the latter could also incorporate a signal of neuronal origin (Liu et al., 2017). Of the 10 regressors we tested, the CSF, global signal, x translation, and x and z rotation were significantly synchronized with the EGG signal (all *p* < .05, FDR corrected; Figure 2a, left). Next, we asked whether EGG‐confounds synchrony might correlate with the extent of brain‐wide PLV with the EGG signal. When these confounds were not included as neural regressors, this correlation was found for the WM, global signal, and x and y rotation (all *p* < .05, FDR corrected. After regressing out these factors from the BOLD signal, none of them were found significant; Figure 2b; see Figures S7–S10 for individual scatter plots). In line with this finding, the confounded BOLD data resulted in a larger difference between the empirical and the null PLV values (*t* = 171.97, *p* < .01; Figure 2d) and a smaller number of significant voxels (confounded: 25504, %19.89 of the GM; cleaned: 1491, %1.16 of the GM; 2 mm^3^ voxels; Figure 2c). All results were reproduced in the CERB data (see Supporting Information 8.4). These results suggest that cleaning non‐GM signals and head motion from the BOLD signal is crucial in order to avoid over‐estimation of brain‐gastric synchrony.

### 3.3 Within‐subject reliability of the EGG and brain signals and the synchrony among them

**FIGURE 3:**
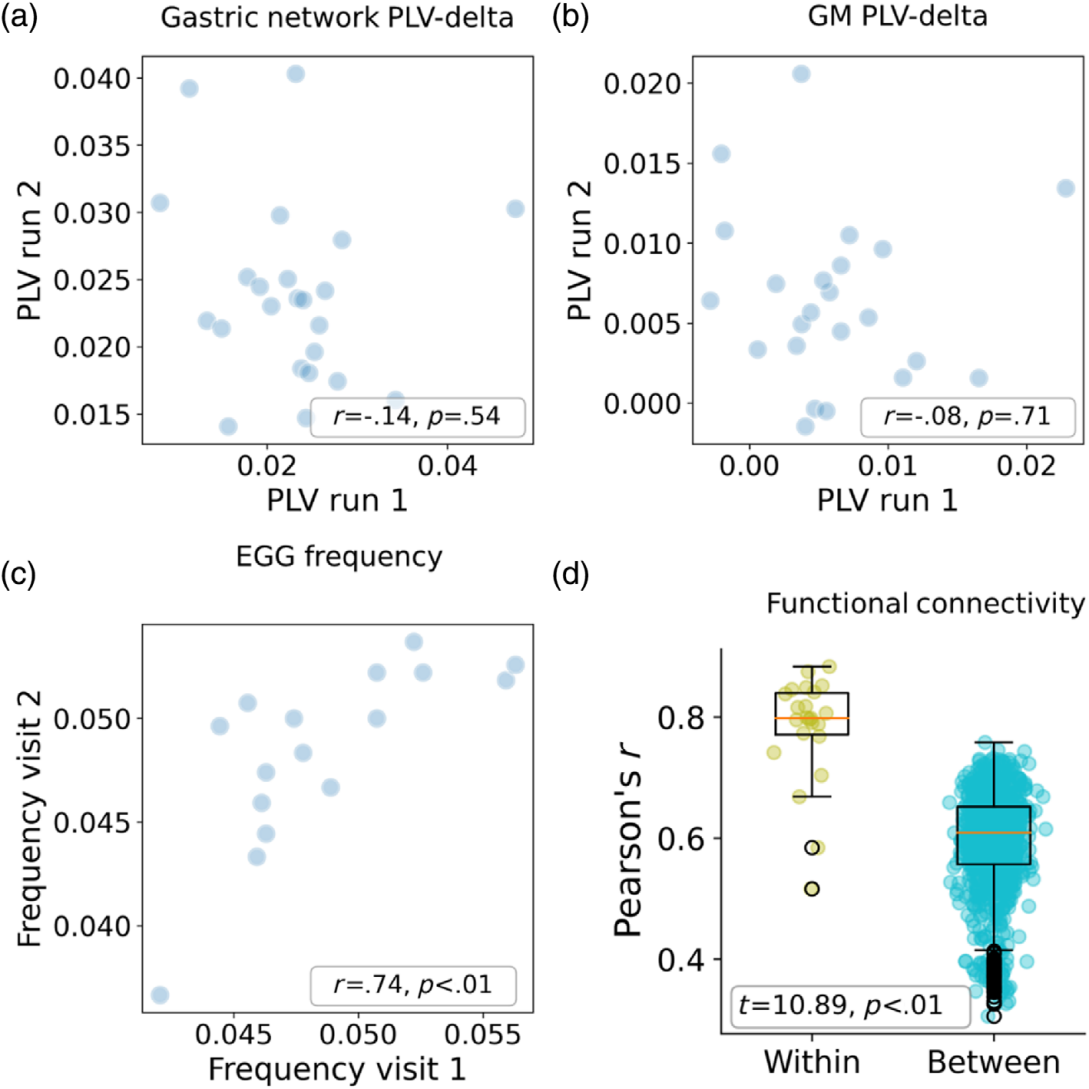
Within‐subject reliability of gastric‐brain synchrony. The correlation of the average PLV‐delta within the significant gastric network voxels (a) or within the GM mask (b) between the first and second runs. (c) The correlation of the peak EGG frequency of the same subject between the lab and MRI recordings. (d) A box plot depicting the correlation of all functional connectivity edges between runs of the same (yellow) and different (blue) subjects.

So far, in two datasets, we found that multiple brain regions exhibited significant synchrony with the EGG signal despite strict confound regression. However, it is still not clear whether the extent of this gastric‐brain synchrony is a reliable within‐subject trait. To estimate within‐subject reliability, we computed the correlation of the PLV‐delta between the first and the second runs across subjects. We did not find such a correlation when measuring the average PLV‐delta within the significant gastric network voxels (*r*(21) = −.14, *p* = .54; Figure 3a) and within the GM mask (*r*(21) = −.08, *p* = .71; Figure 3b). We additionally compared the mean PLV value within each node of Schaefer 100 nodes atlas (Schaefer et al., 2018) across participants as in Müller et al. (2022). We computed the Pearson's correlation of the mean PLVs between all possible participant pairs and found that the correlation was not significantly different from zero (mean = .003, STD = .136; *t*(1033) = 0.762, *p* = .446). To verify that these null results could not be due to low reliability of either the BOLD or the EGG data, we repeated the analysis separately for the peak of the EGG signal and for cortex‐wide functional connectivity values. The peak of the EGG signal was compared between the recordings obtained in the lab and during the MRI scans, and the two were significantly correlated (*r*(14) = .74, *p* < .01; Figure 3c) despite being measured months apart and in two different setups. Functional connectivity reliability was measured as the correlation between all possible edges across runs (Levakov et al., 2021). Functional connectivity presented a significantly higher correlation within subjects compared to between different subjects (*t*(1033) = 10.89, *p* < .01; Figure 3d). We could not repeat these analyses in the CERB dataset since they included data from only a single subject. These findings suggest that brain‐gastric synchrony presents low reliability.

## 4 DISCUSSION

The complex interplay between the stomach and the brain can be assessed by measuring neural‐gastric coupling noninvasively. In the current work, we examined whether previous reports of such coupling (Rebollo et al., 2018) would generalize to an analysis pipeline that included strict control over potential confounds. Moreover, we examined the extent of within‐subject reliability of these results. First, we derived the gastric network following cleaning of the global signal, non‐GM signal, head motion, and cardiac artifacts from the BOLD data. Our gastric network displayed significant spatial similarity to those obtained by Rebollo and Tallon‐Baudry (2022). Next, we found that the global signal, non‐GM fMRI signal and head motion measures were synchronized with the EGG signal. Accordingly, confounds‐EGG synchrony was correlated with the extent of the estimated gastric network. Rigorous confound regression eliminated this correlation, suggesting that confound control is necessary to avoid an overestimation of stomach‐brain coupling. Importantly, all findings, besides the spatial similarity to Rebollo and Tallon‐Baudry (2022), were reproduced in an additional dataset of a highly sampled individual. Finally, we found that while standard EGG and fMRI measures showed substantial test–retest reliability, measures of gastric‐brain synchrony did not.

### 4.1 Motion correction in stomach‐brain coupling estimation

Our results suggest that strict confound control, mainly correcting for head motion, is necessary to avoid an overestimation of gastric‐brain synchrony. Similar results are often found when estimating functional connectivity based on fMRI, where head motion might introduce spurious correlations across brain regions (Power et al., 2014). These motion artifacts cannot be adjusted by simple motion correction due to partial volume effects (Hahamy et al., 2014), spin history effects (Muraskin et al., 2013), and nonrigid motion (Ooi et al., 2013), hence additional steps are typically applied. These steps include regressing out the translation and rotation estimates from the motion correction algorithm, the derivatives, and squares of these motion estimates (Friston et al., 1996), motion censoring (“scrubbing”; Power et al., 2012), and exclusion of subjects with excessive head motion. Finally, subject‐wise summary motion statistics are often used as covariates in the second‐level analysis, and their correlation to any variable of interest is examined. Previous studies did not apply all, or some of these steps (Choe et al., 2021; Müller et al., 2022; Rebollo et al., 2018; Rebollo & Tallon‐Baudry, 2022; see Table S1), and none applied motion regression. The substantial effect of motion we report here suggests that it should be tightly controlled in future studies. One potential mechanism in which motion might affect gastric‐brain coupling is breathing. Respiration affects both head motion (Glover et al., 2000) and the gastric signal (Verhagen et al., 1999), and its frequency (~0.3 Hz) might partially overlap with the EGG signal (Yin & Chen, 2013). However, in the current study, we did not record the respiratory signal or measured abdominal motion hence we could not provide direct evidence supporting this mechanism. A previous study did account for respiratory signal by implementing corrections such as RETROICOR (Müller et al., 2022), providing a partial solution to this issue. However, the effect of motion on EGG and brain MRI is highly complex and nonlinear and, consequently, extremely challenging to model (Corona et al., 2019). In contrast to controlling for motion artifacts, regressing out the global signal as part of the preprocessing procedure is more controversial (Murphy & Fox, 2017). The global signal was found to be correlated with various nuisance variables such as motion parameters and the signal from the cerebrospinal fluid but also with vigilance and arousal levels and electrophysiological recordings of local field potentials and electroencephalogram (Liu et al., 2017). In our work, not including global signal regression resulted in a more widespread activation pattern with greater similarity to previous work (Rebollo & Tallon‐Baudry, 2022).

### 4.2 Stomach‐brain coupling beyond motion artifacts

In the current work, we report the substantial susceptibility of stomach‐brain coupling estimation to motion artifacts. However, similarly to previous studies, we provide evidence that the gastric network is a robust finding that can be observed regardless of motion effects. First, in our sample, we documented the gastric network following confound regression and reported a significant spatial similarity with previous results (Rebollo & Tallon‐Baudry, 2022). The stomach‐brain coupling was found in an additional sample of a highly sampled individual (Choe et al., 2021), although the spatial similarity to previous work was not found. Additionally, recent work in rats reported a network of sensory, motor, and limbic brain regions coupled with the stomach's electrical rhythm (Cao et al., 2022). In this study, rats were head‐fixed, and head motion parameters were regressed out from the BOLD signal and thus could not account for the finding. Moreover, this synchrony was affected by the hunger state of the rats and was significantly reduced following bilateral vagotomy, supporting a causal role for the stomach signaling in generating this synchrony. Finally, this notion was supported by another recent study, where auricular vagus nerve stimulation in humans increased gastric‐brain coupling (Müller et al., 2022). These findings suggest that the gastric‐brain network shows substantial generalizability across studies and validity with respect to its specificity to the gastric signal. Establishing the generalizability and validity of the results will allow future work to explore the functional significance of gastric brain coupling (Levakov et al., 2021), which is currently far from being understood.

### 4.3 Reliability of gastric‐brain coupling, implications, and possible solutions

We report poor reliability of the gastric‐brain synchrony when measured both within the significant gastric network voxels and within the GM mask. This contrasts with substantial test–retest reliability found in standard EGG and fMRI measures. The reliability of a measurement provides an upper bound to the extent of its correlation to additional variables (Goodwin & Leech, 2006). Hence improving the measurements of gastric‐brain coupling is crucial to allow its use as an interoception biomarker (Khalsa & Lapidus, 2016). If the apparent low reliability is partially related to methodological rather than to physiological causes, possible solutions may involve finding alternative synchrony measures or reducing noise from the gastric or brain recordings. Recent advancements in EGG recording techniques could aid in this endeavor. Such advancements include imaging‐guided electrode placement, high‐density EGG (Gharibans et al., 2019), and methods for artifact detection and removal (Gharibans et al., 2018). Hopefully, these and other methodological advancements will improve the reliability of the measurements of brain‐gastric coupling. Yet, while using current methods, relating this coupling to individual differences in physiology or behavior should be done with great caution (Rebollo & Tallon‐Baudry, 2022).

### 4.4 Generalizability of stomach‐brain phase synchronization

In the current work, we demonstrated that previous reports of brain‐wide synchronization with the gastric electrical signal (Rebollo et al., 2018; Rebollo & Tallon‐Baudry, 2022) generalize to different methodologies and to a different population. Importantly, the similarity to previous work was found following strict control of motion artifacts. We adopted the method reported by Rebollo et al. (2018) for estimating the PLV and some of the first and second‐level analyses. However, we note that the current work is not a replication of previous work as there are several methodological differences between the two studies. These include the use of a different preprocessing pipeline, using a high‐pass filter for the EGG instead of a DC recording, and the use of repetitive measures.

### 4.5 Limitations

We consider several limitations of the current study. First, the high exclusion rate, mainly due to poor EGG quality, was compatible with previous work (Wolpert et al., 2020) but still relatively high (29 out of 122 runs, 24%). A possible interaction between the gastric network characteristics and exclusion impedes the generalizability of the results. Generalizability is also affected by inclusion based on a specific BMI range (18–25). This criterion was used in previous work (Wolpert et al., 2020) as EGG quality might be hampered in individuals with high BMI. A second limitation is the lack of causal manipulation. Vagal or stomach manipulation, as done in other studies (Müller et al., 2022), might help establish the specificity of the gastric‐brain network to the stomach activity or signaling. We additionally consider the sample size as a limitation, as even with 63 participants, a previous study was found to be slightly underpowered, while 43 participants were used in the current work. However, the use of repetitive within‐subject samples may still contribute to increasing the statistical power of our work. Finally, tighter control of the hunger state, for example, by supplying a standard meal before the recording (DiFeliceantonio et al., 2018), might reduce the variability in the signal that is associated with hunger level.

## 5 CONCLUSIONS

In the current study, we established the existence of stomach‐brain coupling following strict control of possible confounds. Our results demonstrate the robustness of the gastric network findings, mainly its generalization to different populations and under various data‐cleaning strategies. We also point to its weakness, its susceptibility to non‐neuronal noise, and its low reliability. We advocate that subsequent studies should focus on improving methodological rigor and developing new recording and analysis techniques to improve the reliability of brain gastric coupling measures. Hopefully, the current work will provide a methodological foundation and scaffolding for future research into stomach–brain interactions and interoception in general.

## FUNDING INFORMATION

This work was supported by a grant from the Israeli Science Foundation (ISF) to Galia Avidan (grant number 2274/21).

## Supporting information


**Data S1:** Supporting Information

**Figure S2:** Supporting Information

## Data Availability

The unprocessed data and a python implementation of the analysis and preprocessing steps are available online (https://github.com/GidLev/brain_gastric_synchronization_2023).
