# Supplementary material for: Reliability and validity of brain‐gastric phase synchronization
Source: Hum Brain Mapp. 2023 Aug 1;44(14):4956–66. doi: 10.1002/hbm.26436 (PMC10472921; doi:10.1002/hbm.26436)
Supplement: Supplementary file 1 — Data S1: Supporting Information [file HBM-44-4956-s002.docx]

Supplementary information (SI)


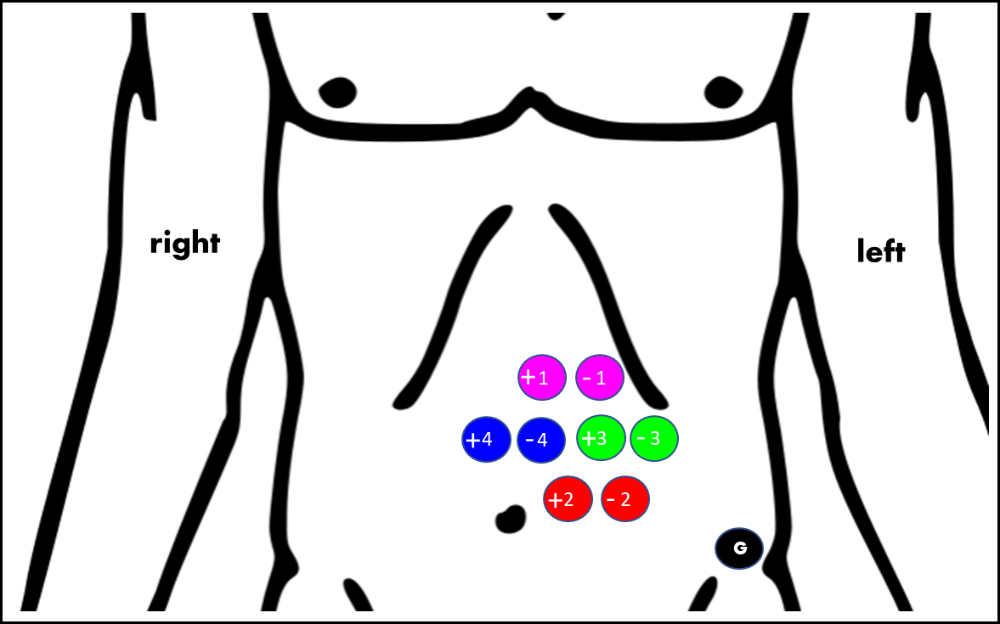


**SI Fig. 1. Electrode placement in accordance with Wolpert et al. (2021). Four bipolar electrodes were placed over the abdomen in three rows. The first electrode pair (±1) were placed 2cm below to midpoint between the xiphoid process and umbilicus with the negative electrode placed just left of the rib cage. The second pair (±2) was positioned 2 cm above the umbilicus, exactly below the first pair. The third and fourth electrode pairs were positioned between pairs 1 and 2 such that the third pair (±3) was placed left of the rib cage and the fourth pair (±4) was centered on the line traversing the xiphoid process and umbilicus. The ground electrode (G) was placed above the iliac crest.**

**SI Fig. 2. EGG spectrogram and assigned quality <Attached as a separate file>. Each subplot depicts the spectrogram of the unprocessed EGG signal. The frequency (Hz) is indicated on the x-axis, and the power spectrum (mV^2^) on the y-axis. The asterisk indicates the selected channel and peak frequency. The color of the title indicates the assigned quality level (1-green, 2-orange, 3-red).**

- 1. *Measuring gastric-brain synchrony and generalization of previous work following motion and cardiac correction*

In this section, we repeated the analysis reported in section 3.1 following the removal of the cardiac signal. The significant clusters are presented on an inflated brain surface and on the cerebellum volume (SI Fig. 3). The significant regions include the bilateral pericalcarine, the right frontal insula, and the pre and post-central gyri. Next, we compared the spatial similarity between our results and those found by Rebollo et al. (2022) using Dice similarity. To estimate the statistical significance of this comparison, we compared our map with 10,000 spatially permuted versions of the Rebollo et al. (2022) map. The empirical similarity was significantly larger than the null distribution (Dice=.015, p=.02).


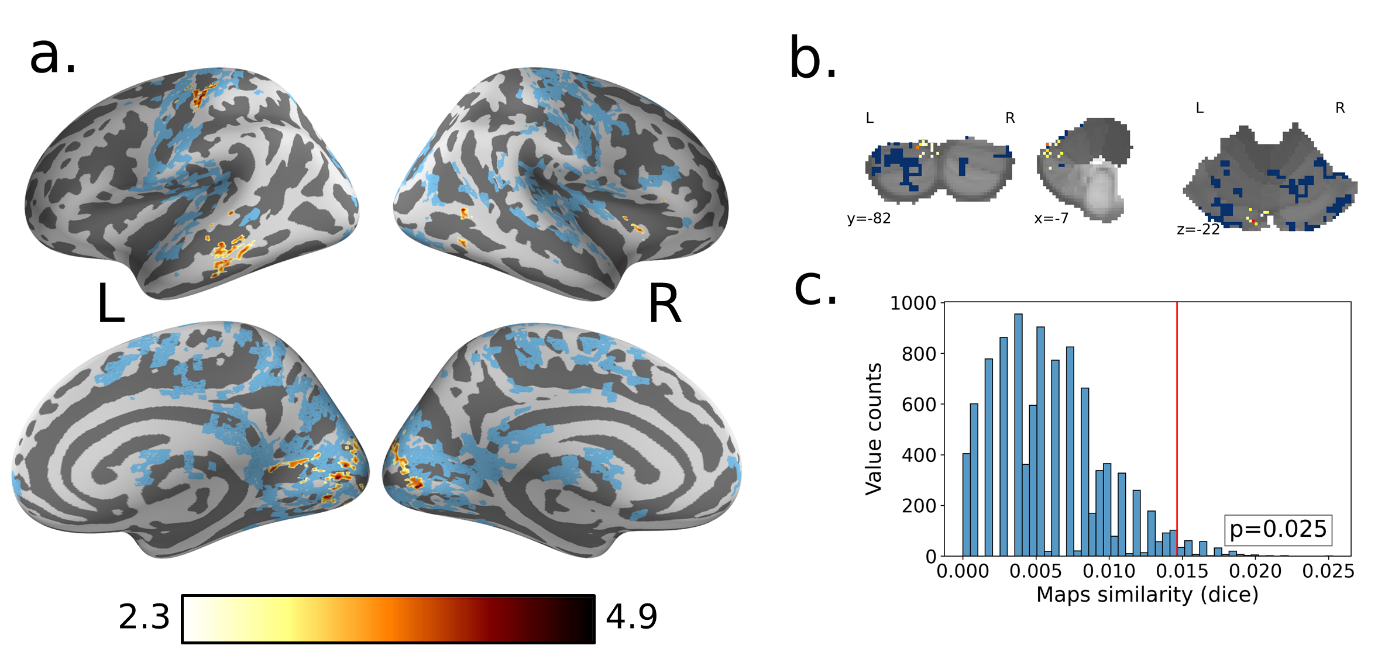


**SI Fig. 3. The gastric-brain network following motion and cardiac correction. The clusters composing the gastric network are depicted on an inflated brain surface (a) and on coronal, sagittal, and axial cerebellum slices (b). Significant clusters from the current study are presented in yellow to red colors representing voxels' t values. Significant clusters from Rebollo et al. (2022) are presented in blue. (c) A histogram of the null distribution of the Dice similarity values obtained from the map of the present study and the spatially permuted map of Rebollo et al. (2022). The vertical red line represents the empirical similarity value.**

- 1. *Measuring gastric-brain synchrony using an alternative null model*

In addition to using a time-shifted version of the stomach signal, we also employed an alternative null model based on the synchrony of each individual’s brain with the gastric signal of the other participants (Choe et al., 2021). The results are presented for the more strict preprocessing version without global signal regression (SI Fig. 4). However, we acknowledge two possible limitations to this method. First, the variability in gastric frequency among subjects (see Fig. 3c) necessitates filtering the brain and stomach signals around the specific gastric peak of each individual (Wolpert et al., 2020), which introduces a positive bias for within-subjects comparison compared to between-subjects comparison. To avoid this, we applied the same filter to all subjects, assuming a constant gastric peak at 0.05Hz. This step might obscure the gastric signal for some participants due to the natural variability in the gastric peak. Secondly, the use of the group as a null distribution resulted in a considerably low number of null samples (Rebollo et al., 2018).

**
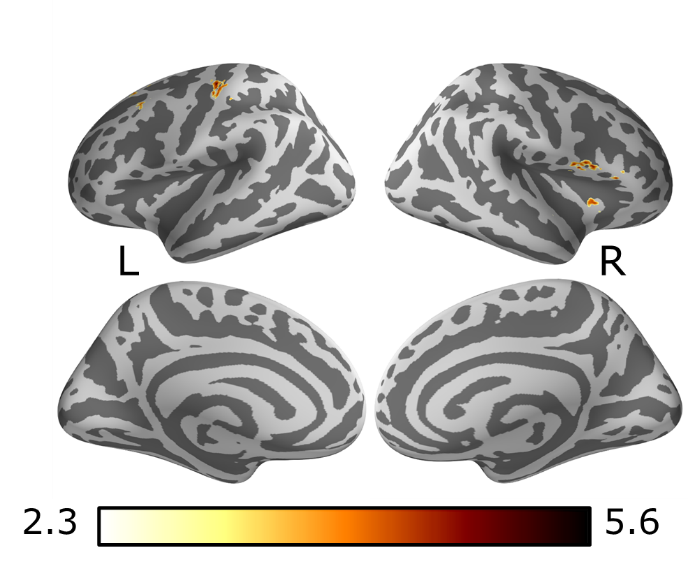
**

**SI Fig. 4. Estimation of the gastric network using an alternative null model. Clusters composing the gastric network are depicted on an inflated brain surface using the other participants as null. The gastric networks are presented in yellow to red colors representing voxels’ t-values.**

- 1. *Measuring gastric-brain synchrony and generalization of previous work following motion correction in the CERB dataset*

This section replicated the results found in section 3.1 in the CERB dataset. The significant clusters are presented on an inflated brain surface (SI Fig. 5). The significant regions include the right precuneus, and paracentral lobules. Next, we compared the spatial similarity between our results and those found by Rebollo et al. (2022) using Dice similarity. To estimate the statistical significance of this comparison, we compared our map with 10,000 spatially permuted versions of the Rebollo et al. (2022) map. The similarity to Rebollo et al. (2022) study was not reproduced (p=.42).


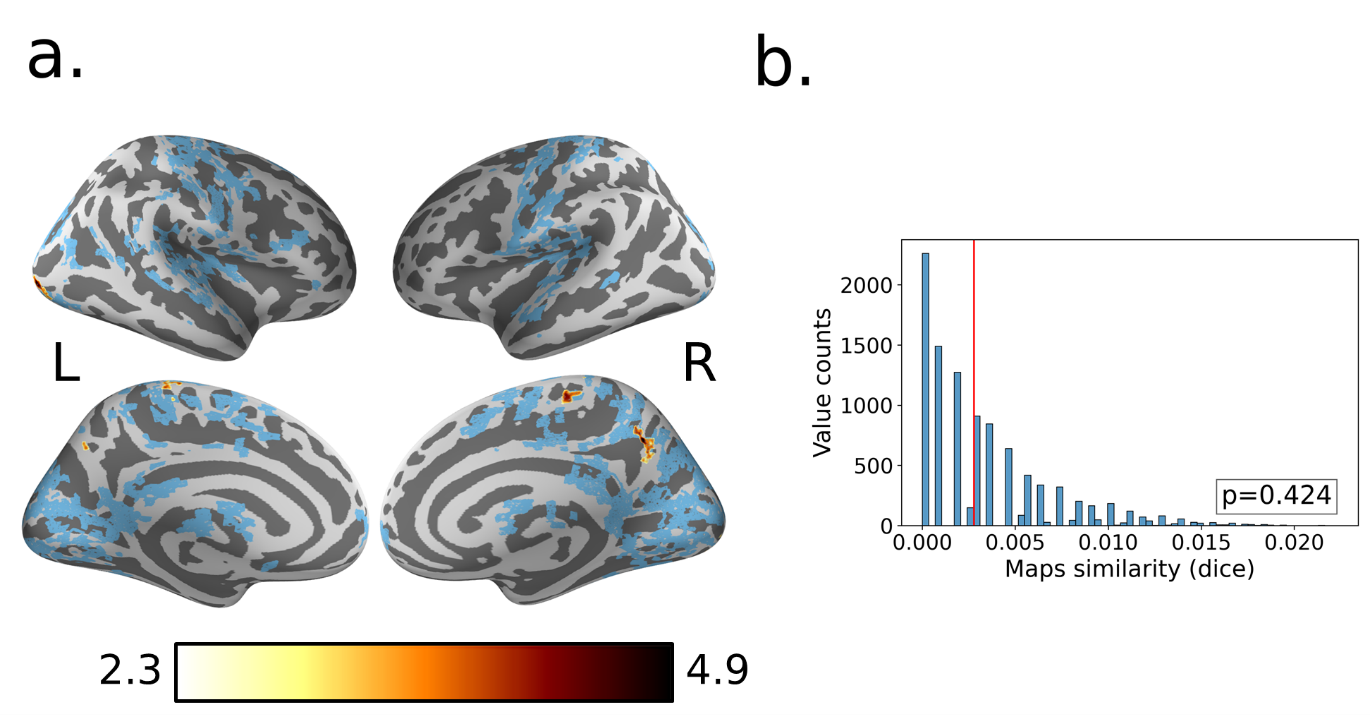


**SI Fig. 5. The gastric-brain network following motion correction in the CERB dataset. (a) The clusters composing the gastric network are depicted on an inflated brain surface. Significant clusters from the current study are presented in yellow to red colors representing voxels' t values. Significant clusters from Rebollo et al. (2022) are presented in blue. (c) A histogram of the null distribution of the Dice similarity values obtained from the map of the present study and the spatially permuted map of Rebollo et al. (2022). The vertical red line represents the empirical similarity value.**

**SI Table. 1. Motion correction strategies in human gastric network papers**

| **Paper** | **N** | **Preprocessing** | **Motion exclusion** | **physiological artifacts correction** | **Motion correction** | **Motion regression** | **Motion 2^nd^ order regression** |
| --- | --- | --- | --- | --- | --- | --- | --- |
| Rebollo et al. 2018 | 30 | SPM | None | RETROICOR (cardiac) | SPM, 6 parameters | None (only CSF) | Mean coupling and FD |
| Rebollo et al. 2022 | 30 + 36 new | SPM | None | RETROICOR (cardiac) | SPM, 6 parameters | None (only CSF) | None |
| Mueller et al. 2021 | 31 | fmriPrep | 1 (>50% of volumes FD > 0.5) | RETROICOR (respiratory) | MCFLIRT | None (only CSF + WM) | None |
| Choe et al. 2021 | 1 (32 sessions) | AFNI | None | None | AFNI | None | None |

- 1. *The relation of motion and additional confounds to brain-gastric synchrony*

In this section, we repeated the analysis included in section 3.2 in the CERB dataset. Of the 10 confounds we tested, the white matter, global signal, x and y translation and x, y and z rotation were significantly synchronized with the EGG signal (all p<0.05, FDR corrected; SI Fig. 6a, left). Next, we asked whether EGG-confounds synchrony might correlate with the extent of brain-wide PLV with the EGG signal. When these confounds were not included as neural regressors, this correlation was found for all confounds except for framewise displacement and x translation and rotations (all p<0.05, FDR corrected). After regressing out these confounds from the BOLD signal, none of the 10 cofounds were found significant (SI Fig. 6b; see SI Fig.8,9 for individual scatter plots). In line with this finding, the confounded BOLD data resulted in a larger difference between the empirical and the null PLV values (t=249.06, p<0.01; Fig. 2d) and a smaller number of significant voxels (confounded: 25257, 19.70% of the GM; cleaned: 306, %0.23 of the GM; 2mm^3^ voxels; SI Fig. 6c).


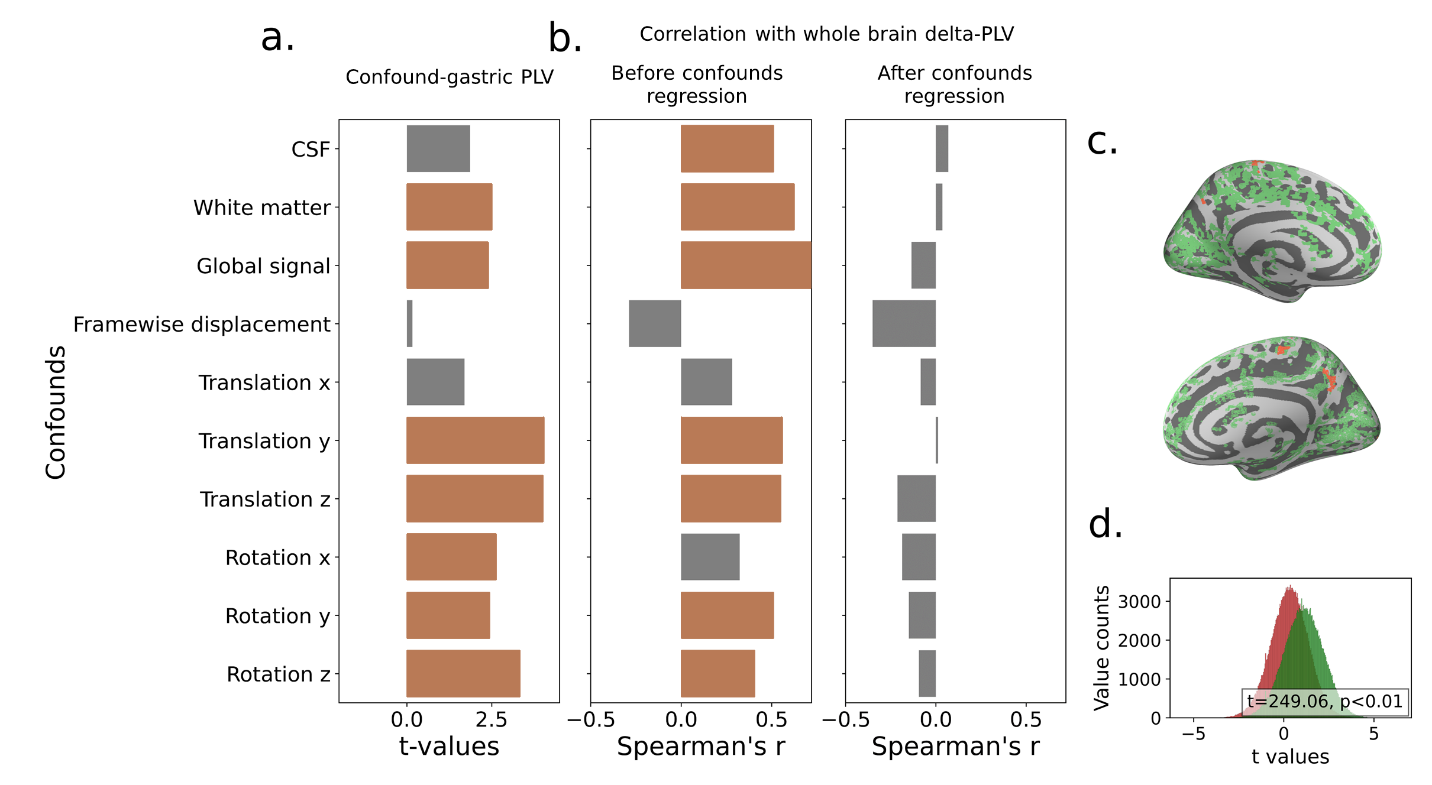


**SI Fig. 6. The effect of various confounds on the gastric-brain estimated synchrony within the CERB dataset. (a) Synchrony between confounds and the EGG signal. Bars represent the paired t-test values between the PLV obtained with the empirical compared to the permuted EGG signal. (b) The correlation of EGG-confounds synchrony with the extent of brain-wide gastric-brain synchrony. The correlations are presented before (left) and after (right) confounds regression from the BOLD signal. In all bar plots, orange-colored bars were significant following a correction for multiple comparisons (p<0.05, FDR corrected). (c) Significant voxels before (green) and after (red) confound regression. (d) The distribution of whole brain t-values before (green) and after (red) confound regression.**


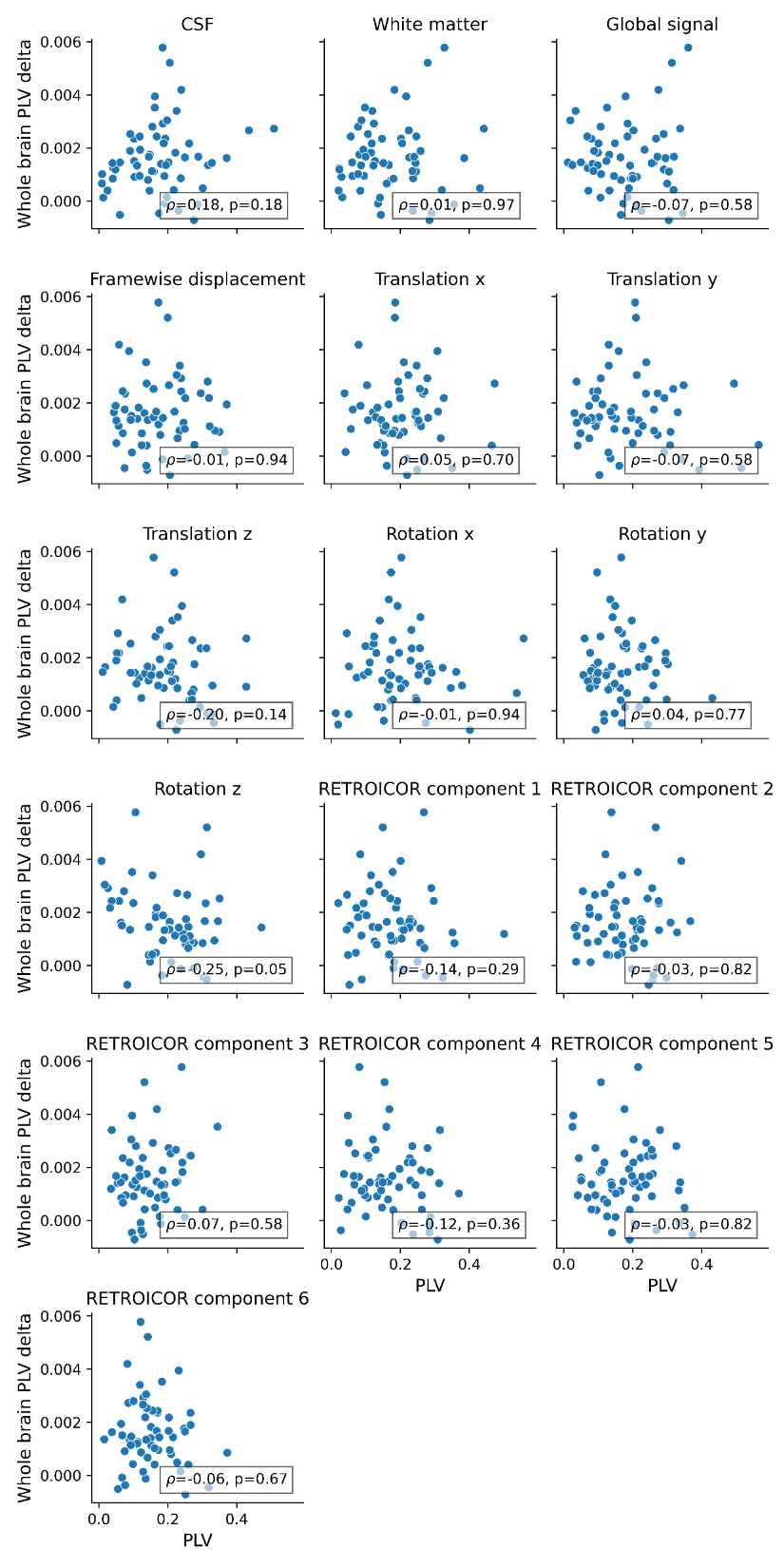


**SI Fig. 7. The relation of EGG-confounds synchrony with the extent of brain-wide PLV with the EGG signal before confound regression. Each subplot depicts EGG-confound PLV (x-axis) compared to whole brain PLV-delta (y-axis). Spearman's correlation and corresponding p-value are indicated in the bottom right of each plot.**


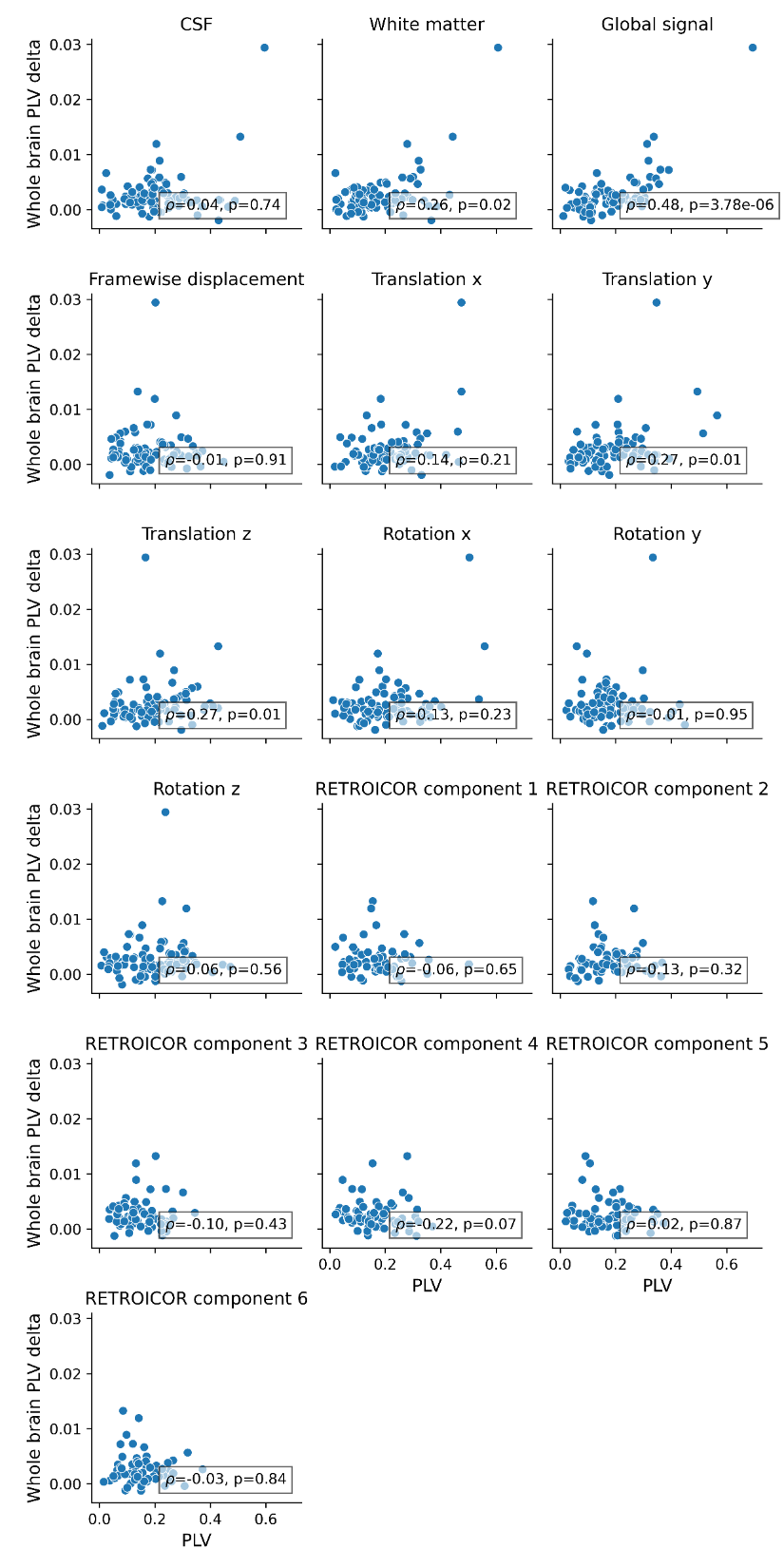


**SI Fig. 8. The relation of EGG-confounds synchrony with the extent of brain-wide PLV with the EGG signal following confound regression. Each subplot depicts EGG-confound PLV (x-axis) compared to whole brain PLV-delta (y-axis). Spearman's correlation and corresponding p-values are indicated in bottom right of each plot.**

**
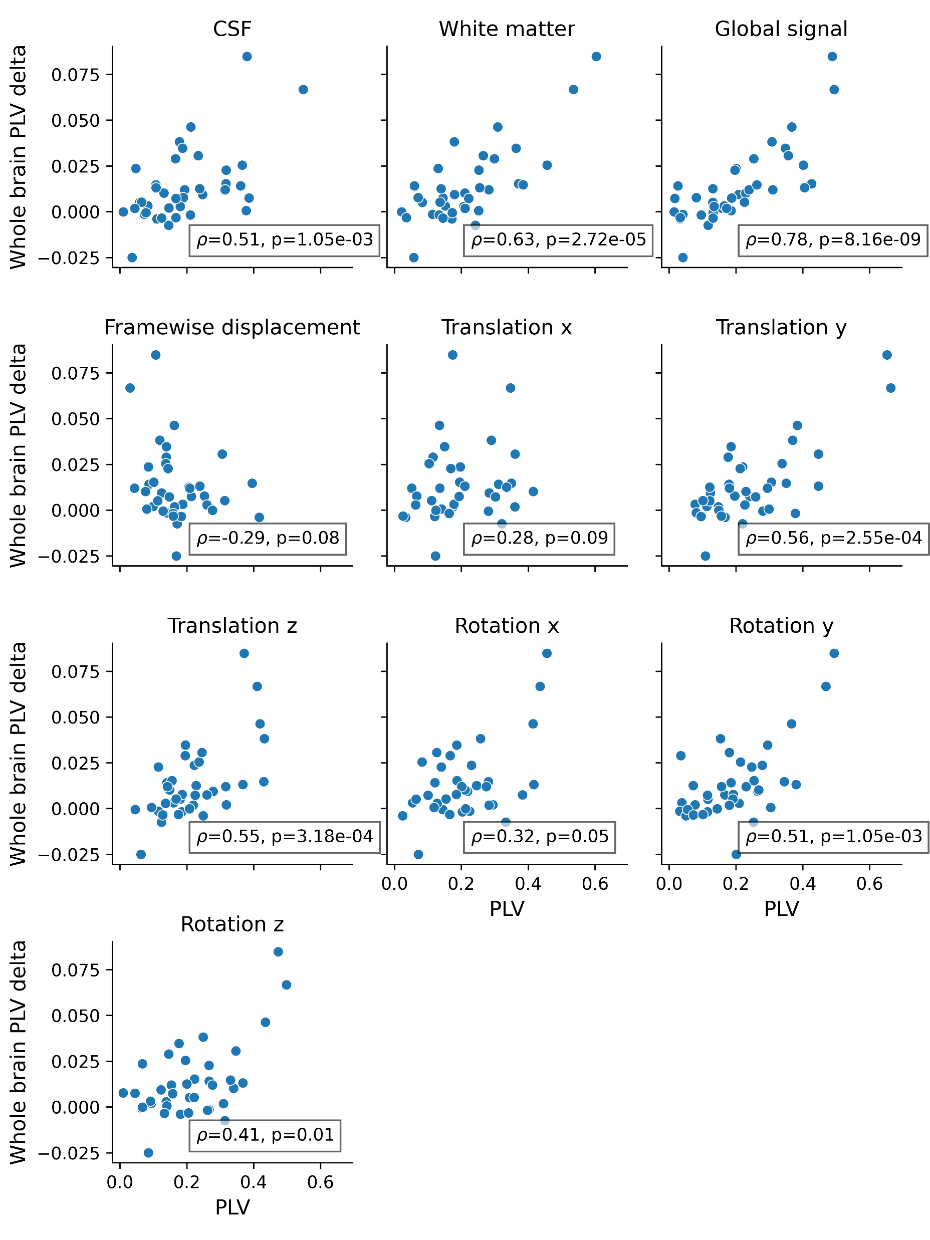
**

**SI Fig. 9. The relation of EGG-confounds synchrony with the extent of brain-wide PLV with the EGG signal before confound regression in the CERB dataset. Each subplot depicts EGG-confound PLV (x-axis) compared to whole brain PLV-delta (y-axis). Spearman's correlation and corresponding p-value are indicated in the bottom right of each plot.**


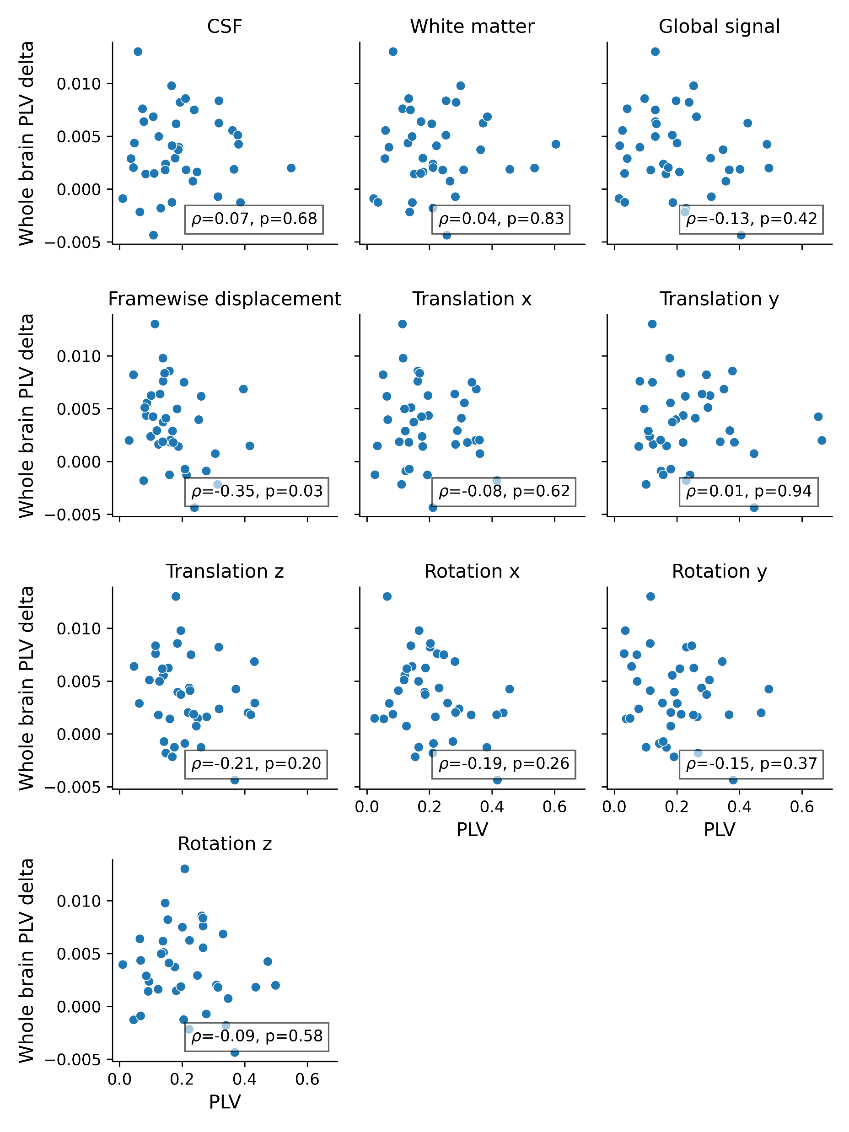


**SI Fig. 10. The relation of EGG-confounds synchrony with the extent of brain-wide PLV with the EGG signal following confound regression in the CERB dataset. Each subplot depicts EGG-confound PLV (x-axis) compared to whole brain PLV-delta (y-axis). Spearman's correlation and corresponding p-value are indicated in the bottom right of each plot.**
